# Genetic diversity and structure of mongolian gazelle (*Procapra gutturosa*) populations in fragmented habitats

**DOI:** 10.1186/s12864-023-09574-0

**Published:** 2023-08-30

**Authors:** Lupeng Shi, Xiufeng Yang, Muha Cha, Tianshu Lyu, Lidong Wang, Shengyang Zhou, Yuehuan Dong, Huashan Dou, Honghai Zhang

**Affiliations:** 1 College of Life Sciences, Qufu Normal University, Qufu, Shandong Province China; 2 Hulunbuir Academy of Inland Lakes in Northern Cold & Arid Areas, Hulunbuir, China

**Keywords:** *Procapra gutturosa*, Genetic diversity, Genetic structure, Microsatellite, Mitochondrial cytochrome b (*Cytb*)

## Abstract

**Background:**

The Mongolian gazelle (*Procapra gutturosa*) population has shown a considerable range of contractions and local extinctions over the last century, owing to habitat fragmentation and poaching. A thorough understanding of the genetic diversity and structure of Mongolian gazelle populations in fragmented habitats is critical for planning effective conservation strategies.

**Result:**

In this study, we used eight microsatellite loci and mitochondrial cytochrome b (*Cytb*) to compare the levels of genetic diversity and genetic structure of Mongolian gazelle populations in the Hulun Lake National Nature Reserve (HLH) with those in the China-Mongolia border area (BJ). The results showed that the nucleotide diversity and observed heterozygosity of the HLH population were lower than those of the BJ population. Moreover, the HLH and BJ populations showed genetic differentiation. We concluded that the HLH population had lower genetic diversity and a distinct genetic structure compared with the BJ population.

**Conclusion:**

The genetic diversity of fragmented Mongolian gazelle populations, can be improved by protecting these populations while reinforcing their gene exchange with other populations. For example, attempts can be made to introduce new individuals with higher genetic diversity from other populations to reduce inbreeding.

**Supplementary Information:**

The online version contains supplementary material available at 10.1186/s12864-023-09574-0.

## Introduction

Globally, severe human disturbances can easily lead to fragmentation of natural landscapes, resulting in fragmentation of the distribution of many animals [1]. Small populations in fragmented habitats are susceptible to factors such as inbreeding and genetic drift, and are often characterized by high extinction rates, low genetic diversity, and declining numbers [2–5]. Therefore, to effectively protect and manage animal populations in fragmented habitats, additional population genetics studies are needed to assess the genetic factors associated with extinction risk [6].

Mongolian gazelles are one of the largest existing wild animal populations in Asia. Over the past 50 years, their distribution area has decreased by approximately 190,000 km^2^, owing to habitat destruction caused by human disturbance and other factors [7, 8]. The present range of the Mongolian gazelle is limited to Mongolia and the adjacent areas of north-eastern China and Russia. High mobility is characteristic of Mongolian gazelle. In BJ, they aggregate throughout the year. The populations of Mongolian gazelle vary in size seasonally due to mating and calving. The recent population estimate is 1,100,000 individuals within a 275,000 km^2^ portion. At present, population size of Mongolian gazelle is approximately 1,000 individuals in China. According to population decline rate and extent of occurrence, Mongolian gazelle is listed as Critically Endangered in China. Protection Category of Mongolian gazelle was listed as a Category I species in the China’s Red List of Biodiversity: Vertebrates [9]. Previous studies on the Mongolian gazelle have mainly focused on macroscopic aspects, such as migration, behavioral characteristics, and feeding habits, and studies on its population genetics are relatively lacking [10–12]. However, an understanding of the genetic diversity of the Mongolian gazelle is important to achieve the optimal conservation of this animal.

The Mongolian gazelle population in HLH is the largest surviving wild population in China. The recent population estimate is 90 individuals within a 14 km^2^ portion. The presence of man-made facilities such as grassland fences precludes gene flow of this Mongolian gazelle population with the outside population. In contrast, the Mongolian gazelle BJ population occupies a contiguous habitat. Therefore, we speculate that the genetic diversity of the fragmented HLH habitat is low, and that it is has genetically differentiated from other populations. This study aimed to explore the genetic diversity and structure of Mongolian gazelle populations in fragmented habitats using microsatellite DNA and *Cytb*. These results will provide a scientific basis for formulating Mongolian gazelle protection strategies.

## Materials and methods

### Sample collection and DNA extraction

**Fig. 1:**
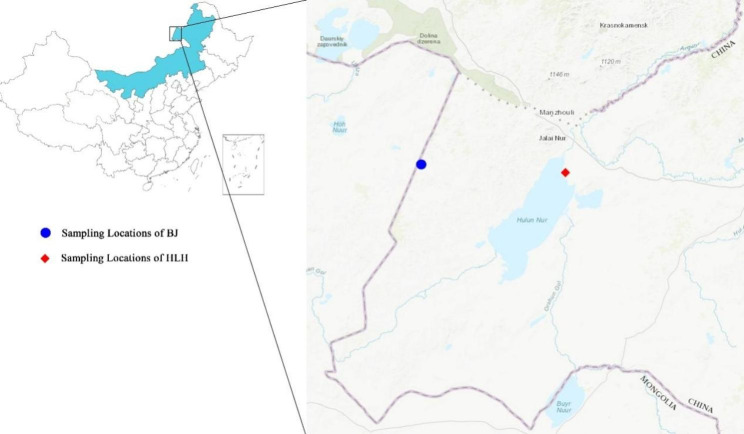
Sampling locations

Fecal samples were collected from the HLH and BJ areas (Fig. 1). The sampling area was grassland covered by snow. Total of 146 samples were collected before consolidation of duplicate animals. Individuals were identified using eight microsatellite loci (OArFCB304, SPS115, TGLA68, IOBT395, PZE114, MNS72, BM1341, and MB066) [13–19]. If all microsatellite sites in the fecal samples had one allele mismatch or all alleles were identical to the genotype, we determined that these fecal samples were from the same Mongolian gazelle [20]. Fecal samples were collected from 54 different individuals after removal of duplicates (Table 1). 27 fecal samples represent 30% of the Mongolian gazelle population in HLH, which is sufficient to represent the overall diversity. According to the China Meteorological Data Service Center (http://data.cma.cn/), the daily average ambient temperature was approximately − 30℃ during the collection time (Table 1) to ensure the quality of DNA. The Mongolian gazelle sample tubes were stored at − 80℃ until DNA extraction. Total genomic DNA was extracted using the QIAamp Fast DNA Stool Mini Kit (Qiagen, Germany), in accordance with the manufacturer’s recommendations.

**Table 1:** Detailed information for all samples

Sample group	Collection location	Sample name	Collection time
HLH	Hulun Lake National Nature Reserve	H1—H27	2018/12 2019/12
BJ	China-Mongolia border area	B1—B27	2019/12

### Microsatellite genotyping and data analysis

The primers were labeled with FAM or HEX fluorescent tags. Polymerase chain reaction (PCR) amplification was performed in a 50 µL solution consisting of 0.3 µL of Taq polymerase, 5.0 µL of 10 × buffer, 4.0 µL of dNTP, 0.3 µL of bovine serum albumin solution, 1.2 µL of each primer, and 6 µL of DNA. Then, H_2_O was added to the PCR mixture to make a final volume of 50 µL. The reactions were performed in a Veriti thermal cycler (Applied Biosystems) with an initial denaturation (95 °C for 5 min); 35 cycles of denaturation (94 °C for 30 s), annealing (50–63 °C for 30 s) and extension (72 °C for 35 s); followed by final extension (72 °C for 7 min). The PCR products were analyzed using an ABI 3730 XL DNA analyzer and GeneMapper (Applied Biosystems). We calculated the number of alleles (Na), number of effective alleles (Ne), observed heterozygosity (Ho), and expected heterozygosity (He) using GenAlEx 6.5 to assess the levels of genetic diversity among populations [21]. We also explored the genetic structures of Mongolian gazelles using the STRUCTURE software [22]. The appropriate number of population clusters (K) was calculated using the STRUCTURE HARVESTE [23]. Finally, we calculated the differentiation among and within populations using analysis of molecular variance (AMOVA) and principal coordinate analysis (PCoA).

### Mitochondrial DNA amplification and data analysis

Due to the fecal quality and other factors, we amplified *Cytb* sequences (1140 bp) of 34 individuals (HLH, 17; BJ, 17) using the primers F- CCCATAGATAGGTGAAGGT and R- CAGGGAATAGTTTAAGCAG. Primers were designed according to the mitochondrial genome sequence of *Procapra przewalskii* (GenBank: MG674218.1) using Primer software [24]. PCR amplification was performed in a 50 µL solution consisting of 0.5 µL of Taq polymerase, 0.5 µL of bovine serum albumin solution, 5.0 µL of 10 × buffer, 4.0 µL of dNTP, 2.0 µL each primer, and 4 µL of DNA. H_2_O was then added to the PCR mixture to make a final volume of 50 µL. The reactions were performed in a Veriti thermal cycler (Applied Biosystems) with an initial denaturation (95 °C for 5 min); 40 cycles of denaturation (94 °C for 45 s), annealing (50 °C for 30 s) and extension (72 °C for 90 s); and a final extension (72 °C for 10 min). PCR products were sequenced using an ABI 3730 XL DNA analyzer. Alignments of the *Cytb* sequences in Mongolian gazelles were performed using MEGA software [25]. DnaSP software was used to analyze genetic diversity indexes, including the number of individuals (N), number of haplotypes (H), haplotype diversity (h), and nucleotide diversity (π) [26]. Finally, haplotype networks of Mongolian gazelles were constructed using the median-joining algorithm in Popart software [27].

## Result

### Microsatellite genetic diversity

Analyses of eight microsatellite loci among the two Mongolian gazelle populations showed a higher mean number of alleles (Na_BJ_ = 7.625 [SD = 3.773]) and observed heterozygosity (Ho_BJ_ = 0.593 [SD = 0.224]) in the samples from BJ than in those from HLH (Na_HLH_ = 5.000 [SD = 1.803]; Ho_HLH_ = 0.523 [SD = 0.171]). The highest number of effective alleles (Ne = 7.010) and expected heterozygosity (He = 0.857) were found at the OArFCB304 locus in the BJ population (Table 2). However, there was no significant difference between HLH and BJ in these values (Na, Ho, Ne, and He). In general, the BJ population showed higher genetic diversity than the HLH population.

**Table 2:** Genetic diversity parameters inferred from 8 microsatellite loci

Population	Locus	Na	Ne	Ho	He
HLH	OArFCB304	5.000	3.163	0.741	0.684
	SPS115	7.000	3.069	0.667	0.674
	TGLA68	3.000	1.774	0.519	0.436
	IOBT395	7.000	5.903	0.778	0.831
	PZE114	7.000	1.913	0.296	0.477
	MNS72	2.000	1.997	0.370	0.499
	BM1341	4.000	1.593	0.407	0.372
	BM066	5.000	1.672	0.407	0.402
	Mean (SD)	5.000 (1.803)	2.636 (1.362)	0.523 (0.171)	0.547 (0.153)
BJ	OArFCB304	10.000	7.010	0.778	0.857
	SPS115	6.000	3.738	0.556	0.733
	TGLA68	2.000	1.246	0.222	0.198
	IOBT395	12.000	6.178	0.741	0.838
	PZE114	6.000	1.486	0.296	0.327
	MNS72	3.000	2.074	0.556	0.518
	BM1341	13.000	6.597	0.926	0.848
	BM066	9.000	4.178	0.667	0.761
	Mean (SD)	7.625 (3.773)	4.063 (2.186)	0.593 (0.224)	0.635 (0.240)

SD: standard deviation; Na: number of alleles; Ne: number of effective alleles; Ho: observed heterozygosity; He: expected heterozygosity

### Mitochondrial genetic diversity

**Fig. 2:**
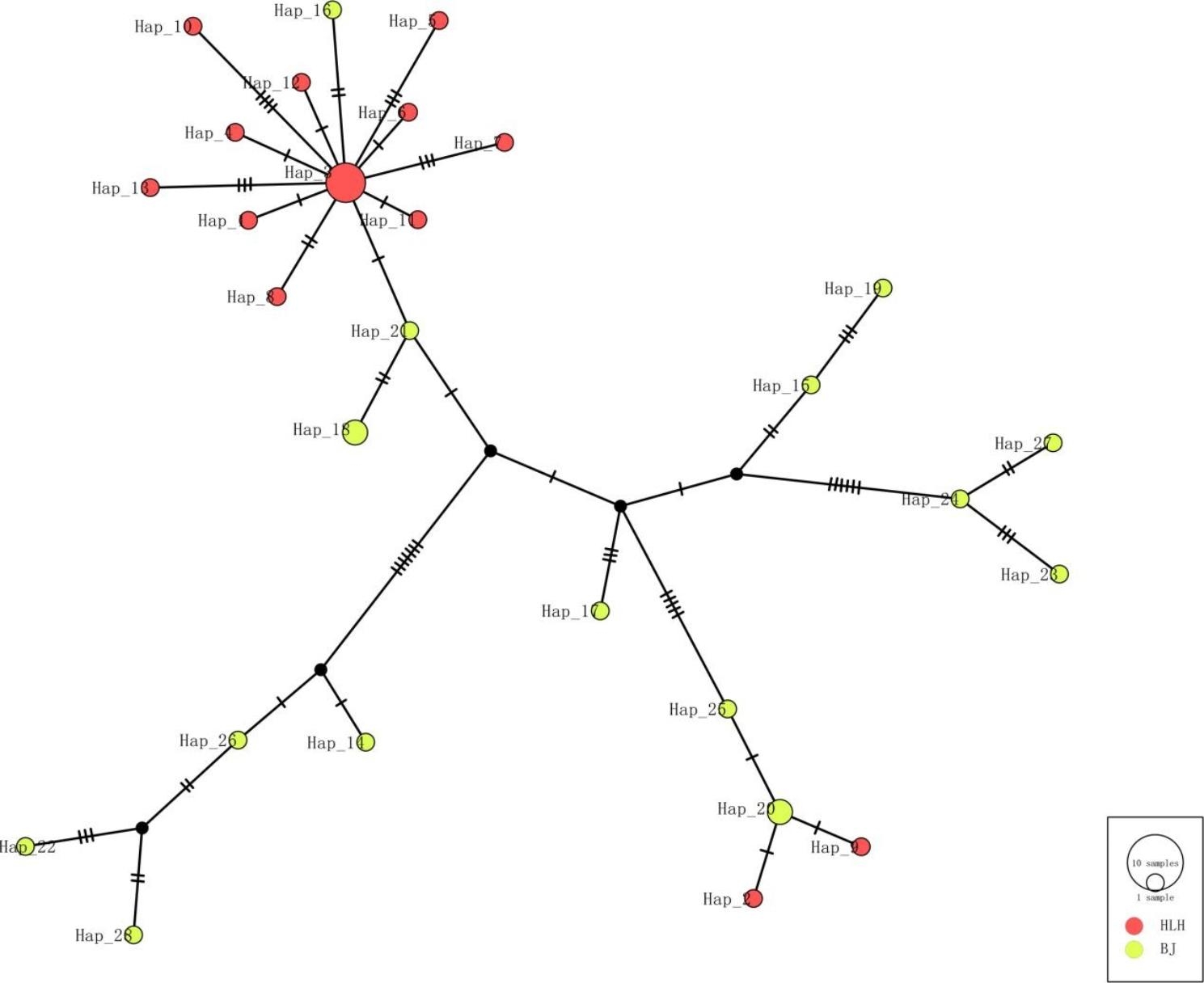
Haplotype network based on *Cytb* sequences. The size of circles represents the number of haplotypes. Different colors represent different populations. The hash lines represent the numbers of mutational steps

The median-joining (MJ) network based on *Cytb* sequences (1140 bp) revealed 28 haplotypes (Fig. 2). Fifteen haplotypes were identified in 17 individuals from BJ, whereas 13 haplotypes were identified in 17 individuals from HLH. Overall, haplotype and nucleotide diversities were high for the BJ population (h = 0.985, π = 0.00992) but lower for the HLH population (h = 0.926, π = 0.00401) (Table 3).

**Table 3:** Genetic diversity parameters inferred from the mitochondrial *Cytb* gene

Population	N	H	h	π
HLH	17	13	0.926	0.00401
BJ	17	15	0.985	0.00992

N: number of individuals; H: number of haplotypes; h: haplotype diversity; π: nucleotide diversity

### Population genetic structure

**Fig. 3:**
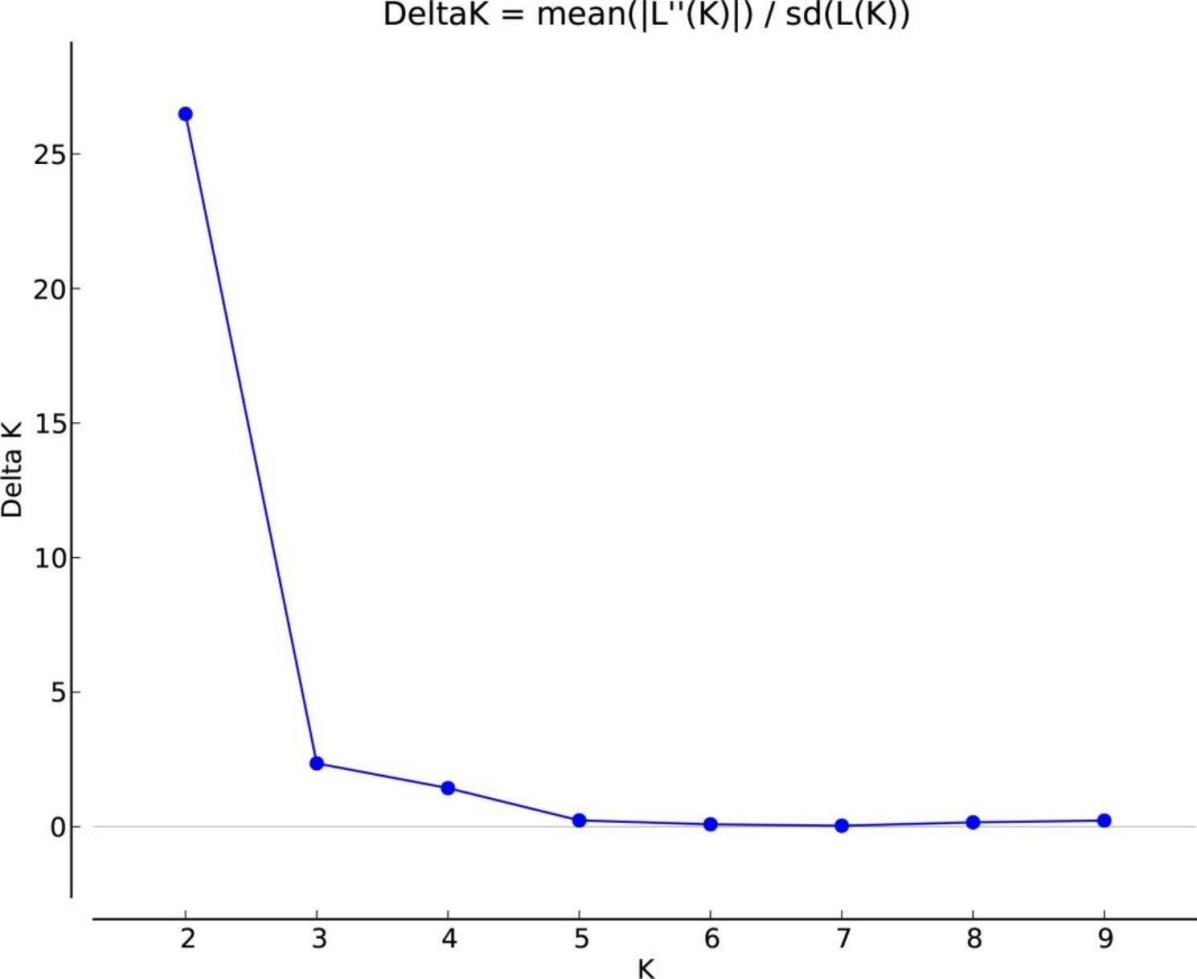
Delta K results. The maximum value was obtained at K = 2

**Fig. 4:**
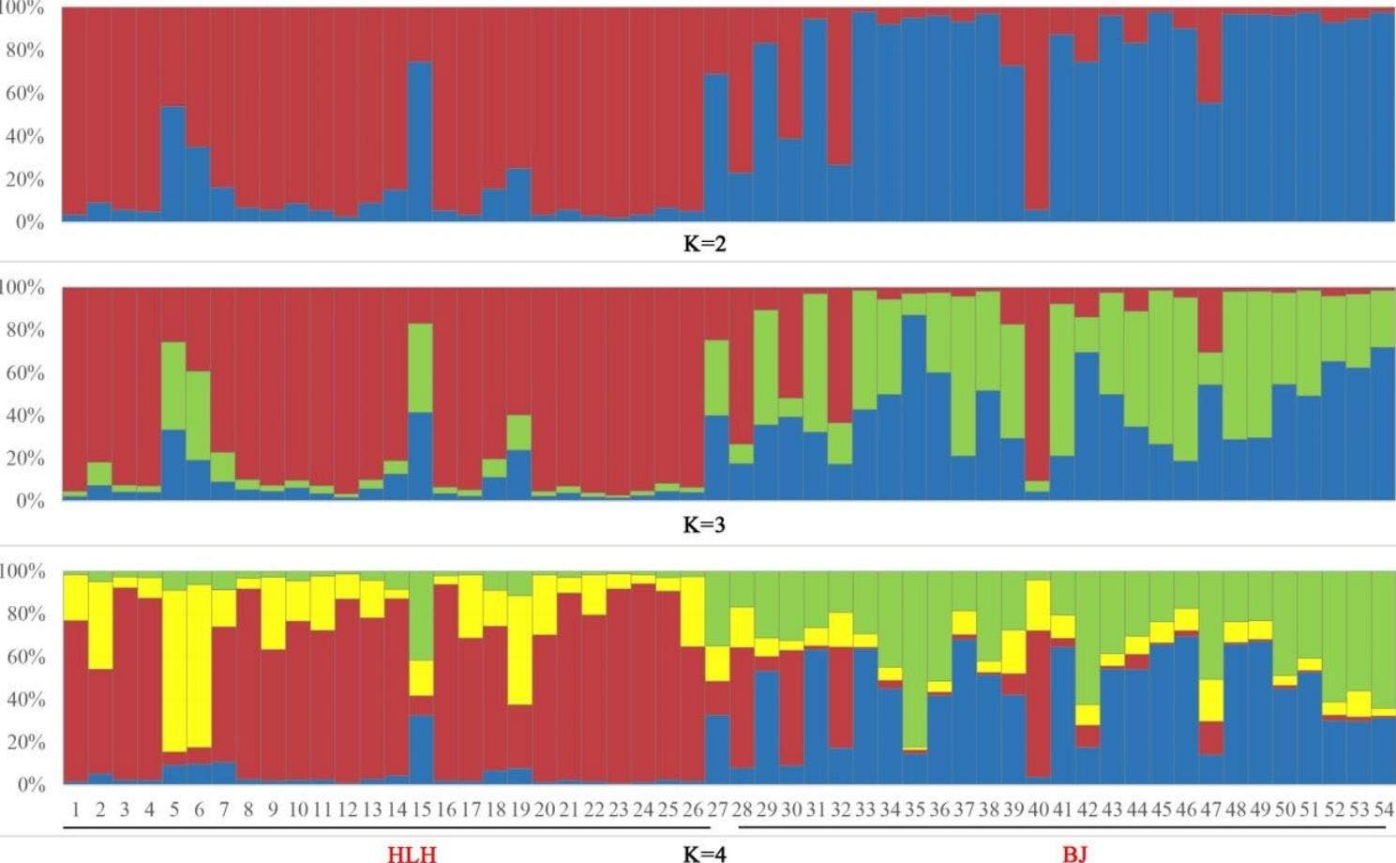
Estimated cluster proportion using STRUCTURE for *Procapra gutturosa*. Each individual is represented by a vertical line

In the STRUCTURE analysis, the delta K values were highest when K = 2, indicating two genetically heterogeneous clusters (Fig. 3). We further plotted the STRUCTURE plots for K = 2, 3, and 4. The findings clearly suggest that the sampled Mongolian gazelle belonged to two large genetic groups: HLH and BJ (Fig. 4).

**Fig. 5:**
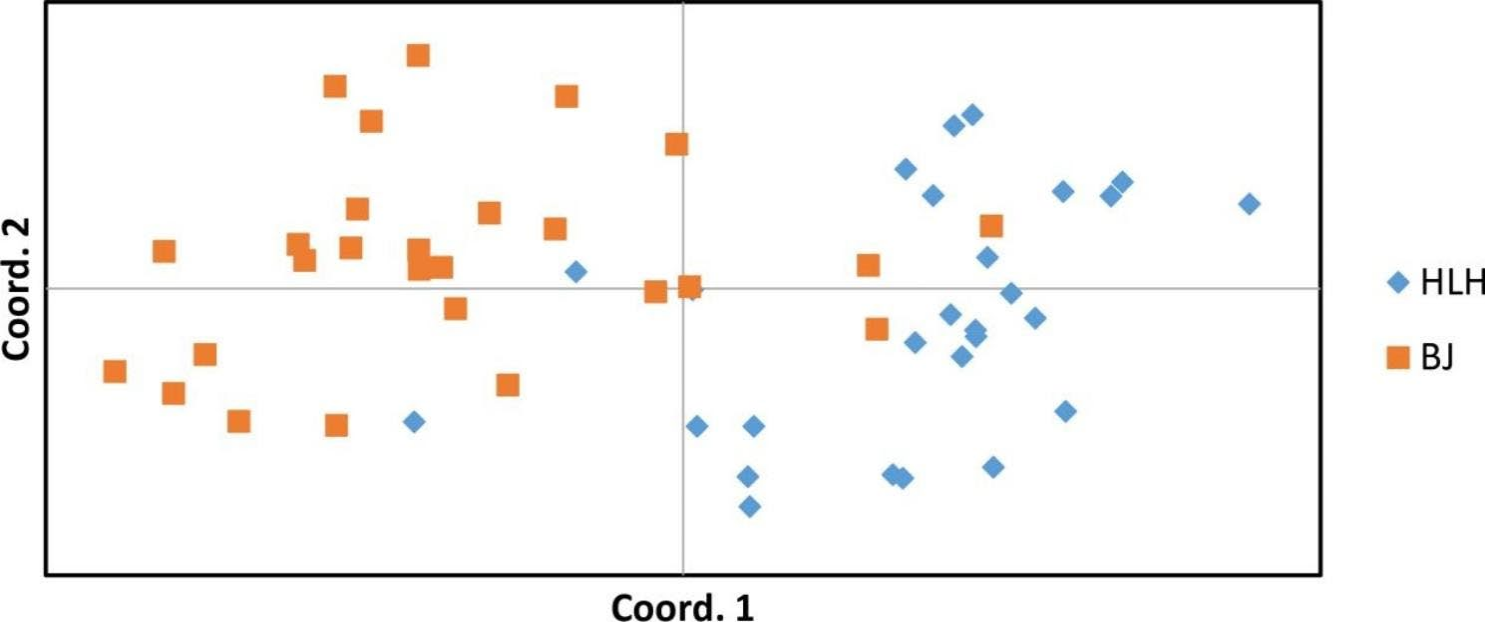
Graph representing Principal Coordinate Analysis (PCoA) of genetic differences among 54 individuals of *Procapra gutturosa*

Consistent with the results of the STRUCTURE analysis, PCoA clustering demonstrated a clear separation between the two populations (Fig. 5). The AMOVA results indicated that most of the genetic variation (81%) occurred within the populations, with only 19% of the variation occurring between populations (Table 4).

**Table 4:** The results of an analysis of molecular variance in genetic partitioning for *Procapra gutturosa* using 8 microsatellite loci

Source of variation	df	Sum of squares	Estimated variance	Percentage of variation (%)
Among Pops	1	37.926	1.213	19%
Within Pops	52	269.593	5.184	81%
Total	53	307.519	6.397	100%

df: degrees of freedom

## Discussion

Genetic diversity affects the adaptation of populations to environmental change [28]. Small populations generally exhibit low genetic variation owing to natural and anthropogenic factors [29]. However, low genetic diversity may increase the vulnerability of small isolated populations to external disturbances, making them more vulnerable to the adverse effects of climate change, disease, and human activities [30]. Thus, low genetic diversity could reduce the survival rate of a species [31]. Therefore, evaluation of the genetic variability of Mongolian gazelle populations in fragmented habitats is important for planning conservation strategies. To assess the genetic diversity and structure of Mongolian gazelles in fragmented habitats, we used microsatellite markers and *Cytb*.

Microsatellite and *Cytb* analyses revealed low levels of genetic diversity in the HLH population, whereas the BJ population exhibited higher levels of genetic diversity. The nucleotide diversity in the HLH population was 0.00401, which was lower than that of the Mongolian gazelle population in a previous study (0.05000) [32]. When the nucleotide diversity of Mongolian gazelles in fragmented habitats was compared with that of other *Procapra* species (*Procapra przewalskii* and *Procapra picticaudata*), it was found to be lower than that of *P. przewalskii* around Qinghai Lake in China (0.01500) and *P. picticaudata* in Tibet (0.08100) [33–35]. In 2015, researchers used mitochondrial and microsatellite markers to study the genetic structure of Mongolian gazelles along the international railroad in Mongolia. These results indicated that the observed heterozygosity of Mongolian gazelles was 0.849 (SD = 0.076) in Mongolia [36]. However, our results indicated that the observed heterozygosity in HLH was 0.523 (SD = 0.171), which was lower than that of the Mongolian gazelle population reported in a previous study. The nucleotide diversity and observed heterozygosity of BJ were 0.00992 and 0.593 (SD = 0.224), respectively, which were higher than those of Mongolian gazelle populations in fragmented habitats, but lower than those in previous studies. These differences may be due to the relatively higher levels of inbreeding in BJ and HLH. Compared to Bovidae species, such as *Moschus moschiferus* (h = 0.970, π = 0.0265) and *Cervus nippon* (h = 0.932, π = 0.0106), the HLH population showed a low level of genetic diversity (h = 0.926, π = 0.00401) [37, 38]. The HLH population also exhibited lower nucleotide diversity than other ungulate species (*Antilope cervicapra*, π = 0.00704; *Cervus elaphus hanglu*, π = 0.008); however, the BJ population exhibited higher nucleotide diversity (h = 0.985, π = 0.00992) than other ungulate species [39, 40]. As such, our results provide strong evidence that the Mongolian gazelle populations in fragmented habitats (HLH) suffer from a loss of genetic diversity. The relatively high level of genetic diversity in the BJ population may be due to gene flow, whereas the HLH population lost its connectivity with other Mongolian gazelle populations. For example, the major Indian *Axis porcinus* population may have been due to historical gene flow, which guarantees a high level of genetic diversity [41]. The low genetic diversity of Mongolian gazelle populations in fragmented habitats may be due to their small population size and the impact of inbreeding. Inbreeding small populations can cause morphological defects in organisms that can lead to the death of these individuals. For example, the inbreeding of Isle Royale wolves has led to some individual skeletal deformities, and more seriously, led this population to the verge of extinction [42]. The ability of a species to maintain its genetic diversity is often essential for ensuring long-term persistence [43]. Populations that lack genetic diversity may show a heightened risk of extinction, owing to their reduced adaptive capacity [44, 45]. Therefore, the establishment of regulations and management schemes for Mongolian gazelle populations in fragmented habitats is urgently required.

In addition to the reduced genetic diversity, our findings showed evidence of genetic differentiation between the HLH and BJ populations. Both PCoA and STRUCTURE analyses demonstrated a clear delineation between the two Mongolian gazelle populations on the basis of allelic composition, implying genetic isolation. Previous studies have shown that natural landscapes, such as mountains, rivers and deserts can act as ecological barriers to gene flow between populations [46–48]. In addition, human activities can affect gene flow between animals [49–52]. In summary, both natural landscapes and human activities have important effects on the genetic structure of populations. For example, the North Chinese leopard populations on the Loess Plateau shows obvious genetic differentiation due to the influence of geographical factors such as the Loess Plateau [53]. We also speculate that the genetic differences may be attributable to a lack of connectivity between Mongolian gazelle populations, small population sizes, and limited dispersal. For example, Isle Royale Moose lacks connectivity with the outside world, leading to genetic differentiation from other moose populations [54]. The AMOVA results indicated that most of the genetic variation was attributed to populations, which further indicated that habitat fragmentation caused by human and natural factors resulted in low genetic diversity within the Mongolian gazelle population [55]. However, as is the case for other species (white-tailed eagles, black rhinoceros, and greater one-horned rhinoceros), considerable genetic diversity is retained within small populations [56–59], indicating the need to strengthen the protection of Mongolian gazelle populations in fragmented habitats.

## Conclusions

We estimated the genetic diversity and structure of Mongolian gazelle populations in fragmented habitats by using microsatellites and *Cytb*. In comparison with the BJ population, the HLH population showed lower genetic diversity and a distinct genetic structure. Low genetic diversity and small population sizes increase the risk of extinction of Mongolian gazelle populations in fragmented habitats. Therefore, increasing the population size and preventing inbreeding are crucial for protection of Mongolian gazelles. Our findings indicate the need for long-term program to monitor the dynamics of the Mongolian gazelle. In addition, for long-term survival of the HLH population, a certain number of Mongolian gazelles should be introduced from other populations.

### Electronic supplementary material

Below is the link to the electronic supplementary material.

Supplementary Material 1

## Data Availability

The mitochondrial *Cytb* genes are available at GenBank repository (https://www.ncbi.nlm.nih.gov/), under the accession number: OP204753–OP204786. The raw data of microsatellite loci is available in Table S1.
