# Supplementary material for: Genetic diversity and structure of mongolian gazelle (Procapra gutturosa) populations in fragmented habitats
Source: BMC Genomics. 2023 Aug 30;24:507. doi: 10.1186/s12864-023-09574-0 (PMC10469424; doi:10.1186/s12864-023-09574-0)
Supplement: Supplementary file 1 — Supplementary Material 1 [file 12864_2023_9574_MOESM1_ESM.docx]

**Table S1**. The raw data of microsatellite loci.

| Sample | OArFCB304 | OArFCB304 | SPS115 | SPS115 | TGLA68 | TGLA68 | IOBT395 | IOBT395 |
| --- | --- | --- | --- | --- | --- | --- | --- | --- |
| H1 | 141 | 145 | 252 | 254 | 81 | 81 | 90 | 90 |
| H2 | 139 | 139 | 252 | 252 | 81 | 81 | 82 | 94 |
| H3 | 145 | 145 | 248 | 256 | 81 | 81 | 82 | 82 |
| H4 | 139 | 141 | 248 | 252 | 81 | 81 | 82 | 94 |
| H5 | 139 | 139 | 252 | 252 | 75 | 75 | 92 | 92 |
| H6 | 139 | 139 | 252 | 252 | 81 | 81 | 92 | 92 |
| H7 | 141 | 145 | 252 | 254 | 81 | 81 | 82 | 90 |
| H8 | 141 | 145 | 252 | 258 | 81 | 111 | 90 | 94 |
| H9 | 141 | 145 | 252 | 260 | 81 | 81 | 98 | 98 |
| H10 | 141 | 145 | 252 | 256 | 81 | 111 | 90 | 94 |
| H11 | 141 | 145 | 254 | 256 | 81 | 81 | 90 | 98 |
| H12 | 141 | 145 | 252 | 252 | 81 | 111 | 88 | 94 |
| H13 | 141 | 145 | 252 | 254 | 81 | 111 | 90 | 94 |
| H14 | 141 | 145 | 252 | 256 | 81 | 111 | 82 | 94 |
| H15 | 139 | 145 | 252 | 256 | 81 | 111 | 92 | 94 |
| H16 | 131 | 141 | 254 | 258 | 81 | 81 | 82 | 94 |
| H17 | 141 | 145 | 252 | 252 | 81 | 81 | 88 | 94 |
| H18 | 141 | 145 | 252 | 252 | 81 | 111 | 88 | 84 |
| H19 | 141 | 141 | 246 | 246 | 81 | 81 | 88 | 92 |
| H20 | 141 | 145 | 252 | 256 | 81 | 111 | 88 | 98 |
| H21 | 131 | 141 | 254 | 256 | 81 | 111 | 88 | 94 |
| H22 | 141 | 141 | 252 | 256 | 81 | 111 | 82 | 88 |
| H23 | 145 | 145 | 252 | 252 | 81 | 81 | 88 | 98 |
| H24 | 131 | 145 | 252 | 258 | 81 | 111 | 82 | 82 |
| H25 | 131 | 145 | 252 | 256 | 81 | 111 | 88 | 94 |
| H26 | 141 | 145 | 252 | 260 | 81 | 111 | 88 | 98 |
| H27 | 135 | 141 | 254 | 254 | 81 | 111 | 90 | 98 |
| B1 | 145 | 145 | 252 | 252 | 81 | 81 | 88 | 94 |
| B2 | 141 | 141 | 254 | 256 | 81 | 81 | 94 | 106 |
| B3 | 133 | 141 | 250 | 252 | 81 | 81 | 82 | 88 |
| B4 | 139 | 141 | 252 | 258 | 81 | 81 | 94 | 106 |
| B5 | 131 | 145 | 252 | 252 | 81 | 81 | 90 | 94 |
| B6 | 137 | 151 | 254 | 254 | 81 | 81 | 100 | 106 |
| B7 | 129 | 131 | 252 | 252 | 81 | 111 | 90 | 100 |
| B8 | 137 | 137 | 256 | 256 | 81 | 81 | 82 | 88 |
| B9 | 131 | 141 | 254 | 254 | 81 | 81 | 86 | 102 |
| B10 | 129 | 129 | 254 | 256 | 81 | 81 | 88 | 92 |
| B11 | 139 | 147 | 254 | 258 | 81 | 111 | 84 | 94 |
| B12 | 135 | 145 | 252 | 252 | 81 | 81 | 94 | 94 |
| B13 | 139 | 141 | 252 | 252 | 81 | 111 | 88 | 98 |
| B14 | 129 | 141 | 252 | 252 | 81 | 81 | 94 | 98 |
| B15 | 139 | 141 | 250 | 250 | 81 | 111 | 88 | 98 |
| B16 | 129 | 147 | 254 | 256 | 81 | 81 | 88 | 100 |
| B17 | 145 | 147 | 254 | 256 | 81 | 81 | 94 | 94 |
| B18 | 129 | 129 | 254 | 256 | 81 | 81 | 92 | 92 |
| B19 | 129 | 141 | 252 | 252 | 81 | 81 | 94 | 94 |
| B20 | 139 | 141 | 252 | 260 | 81 | 81 | 82 | 88 |
| B21 | 129 | 129 | 254 | 256 | 81 | 81 | 88 | 92 |
| B22 | 139 | 151 | 252 | 254 | 81 | 81 | 94 | 106 |
| B23 | 129 | 139 | 254 | 258 | 81 | 111 | 94 | 94 |
| B24 | 139 | 147 | 254 | 258 | 81 | 81 | 84 | 92 |
| B25 | 139 | 141 | 252 | 252 | 81 | 111 | 94 | 108 |
| B26 | 131 | 135 | 252 | 256 | 81 | 81 | 92 | 92 |
| B27 | 131 | 147 | 250 | 254 | 81 | 81 | 92 | 92 |
| Sample | PZE114 | PZE114 | MNS72 | MNS72 | BM1341 | BM1341 | BM066 | BM066 |
| H1 | 93 | 93 | 164 | 166 | 120 | 120 | 100 | 100 |
| H2 | 93 | 93 | 164 | 164 | 120 | 120 | 96 | 100 |
| H3 | 93 | 93 | 164 | 164 | 116 | 120 | 100 | 100 |
| H4 | 83 | 93 | 164 | 164 | 120 | 120 | 100 | 100 |
| H5 | 93 | 93 | 166 | 166 | 120 | 120 | 96 | 100 |
| H6 | 93 | 93 | 166 | 166 | 120 | 120 | 96 | 100 |
| H7 | 93 | 93 | 164 | 166 | 120 | 120 | 96 | 98 |
| H8 | 91 | 91 | 164 | 164 | 116 | 120 | 96 | 100 |
| H9 | 93 | 93 | 164 | 166 | 120 | 120 | 100 | 110 |
| H10 | 77 | 93 | 164 | 166 | 118 | 120 | 100 | 100 |
| H11 | 93 | 93 | 166 | 166 | 118 | 120 | 100 | 100 |
| H12 | 91 | 91 | 166 | 166 | 118 | 120 | 100 | 100 |
| H13 | 93 | 111 | 164 | 166 | 120 | 120 | 100 | 100 |
| H14 | 85 | 91 | 164 | 164 | 116 | 120 | 96 | 100 |
| H15 | 93 | 93 | 164 | 166 | 120 | 124 | 98 | 122 |
| H16 | 91 | 91 | 164 | 166 | 120 | 120 | 100 | 100 |
| H17 | 93 | 93 | 166 | 166 | 118 | 120 | 100 | 100 |
| H18 | 81 | 93 | 164 | 166 | 120 | 120 | 96 | 100 |
| H19 | 93 | 93 | 166 | 166 | 118 | 120 | 100 | 100 |
| H20 | 93 | 93 | 166 | 166 | 118 | 120 | 100 | 100 |
| H21 | 83 | 93 | 164 | 166 | 120 | 120 | 100 | 100 |
| H22 | 93 | 93 | 166 | 166 | 120 | 120 | 100 | 100 |
| H23 | 83 | 91 | 164 | 164 | 120 | 120 | 100 | 100 |
| H24 | 93 | 93 | 164 | 164 | 120 | 120 | 100 | 100 |
| H25 | 81 | 93 | 164 | 164 | 120 | 120 | 100 | 100 |
| H26 | 93 | 93 | 164 | 166 | 120 | 120 | 100 | 110 |
| H27 | 93 | 93 | 166 | 166 | 116 | 118 | 98 | 100 |
| B1 | 93 | 93 | 164 | 166 | 112 | 128 | 100 | 100 |
| B2 | 93 | 93 | 164 | 166 | 112 | 116 | 98 | 100 |
| B3 | 91 | 91 | 164 | 166 | 116 | 126 | 100 | 100 |
| B4 | 93 | 93 | 164 | 166 | 114 | 116 | 98 | 98 |
| B5 | 93 | 93 | 164 | 166 | 118 | 122 | 96 | 100 |
| B6 | 93 | 93 | 164 | 166 | 110 | 116 | 98 | 98 |
| B7 | 81 | 93 | 164 | 164 | 122 | 122 | 96 | 98 |
| B8 | 93 | 93 | 154 | 164 | 121 | 128 | 102 | 106 |
| B9 | 93 | 93 | 164 | 166 | 116 | 124 | 96 | 96 |
| B10 | 93 | 93 | 166 | 166 | 116 | 124 | 98 | 100 |
| B11 | 93 | 101 | 164 | 164 | 122 | 124 | 96 | 98 |
| B12 | 93 | 93 | 166 | 166 | 120 | 132 | 98 | 98 |
| B13 | 93 | 93 | 164 | 164 | 116 | 120 | 100 | 100 |
| B14 | 93 | 93 | 164 | 166 | 116 | 124 | 96 | 98 |
| B15 | 93 | 93 | 164 | 164 | 116 | 120 | 102 | 102 |
| B16 | 93 | 93 | 164 | 166 | 114 | 116 | 96 | 110 |
| B17 | 93 | 93 | 164 | 166 | 116 | 132 | 98 | 100 |
| B18 | 81 | 93 | 166 | 166 | 116 | 126 | 96 | 98 |
| B19 | 93 | 93 | 164 | 166 | 116 | 122 | 96 | 98 |
| B20 | 93 | 93 | 164 | 166 | 114 | 130 | 96 | 100 |
| B21 | 93 | 93 | 166 | 166 | 116 | 124 | 94 | 96 |
| B22 | 93 | 93 | 166 | 166 | 110 | 116 | 98 | 98 |
| B23 | 93 | 113 | 164 | 166 | 118 | 124 | 98 | 102 |
| B24 | 93 | 101 | 164 | 164 | 122 | 124 | 96 | 98 |
| B25 | 81 | 93 | 164 | 166 | 114 | 116 | 86 | 98 |
| B26 | 93 | 111 | 166 | 166 | 118 | 118 | 98 | 124 |
| B27 | 81 | 93 | 164 | 164 | 110 | 116 | 102 | 124 |
